# De-tabooing dying control - a grounded theory study

**DOI:** 10.1186/1472-684X-12-13

**Published:** 2013-03-13

**Authors:** Hans O Thulesius, Helen Scott, Gert Helgesson, Niels Lynöe

**Affiliations:** 1 Department of Clinical Sciences Malmö, Division of Family Medicine, Lund University, Lund, Sweden; 2 Research and Development Centre, Kronoberg County Council, Box 1223, SE-351 12, Växjö, Sweden; 3 Grounded Theory Online, PO Box 680, Chichester, West Sussex, UK; 4 Stockholm Centre for Healthcare Ethics, Dept. of Learning, Informatics, Management and Ethics, Karolinska Institutet, Stockholm, Sweden

**Keywords:** Taboo, Death, Dying, Euthanasia, Physician assisted suicide, Palliative care, Sedation therapy, Grounded theory

## Abstract

**Background:**

Dying is inescapable yet remains a neglected issue in modern health care. The research question in this study was “what is going on in the field of dying today?” What emerged was to eventually present a grounded theory of control of dying focusing specifically on how people react in relation to issues about euthanasia and physician-assisted suicide (PAS).

**Methods:**

Classic grounded theory was used to analyze interviews with 55 laypersons and health care professionals in North America and Europe, surveys on attitudes to PAS among physicians and the Swedish general public, and scientific literature, North American discussion forum websites, and news sites.

**Results:**

Open awareness of the nature and timing of a patient’s death became common in health care during the 1960s in the Western world. *Open dying awareness contexts* can be seen as the start of a weakening of a taboo towards controlled dying called *de-tabooing*. The growth of the hospice movement and palliative care, but also the legalization of euthanasia and PAS in the Benelux countries, and PAS in Montana, Oregon and Washington further represents de-tabooing dying control. An *attitude positioning* between the taboo of dying control and a growing taboo against questioning patient autonomy and self-determination called *de-paternalizing* is another aspect of de-tabooing. When confronted with a taboo, people first react emotionally based on “gut feelings” - *emotional positioning*. This is followed by reasoning and label wrestling using euphemisms and dysphemisms - *reflective positioning*. Rarely is de-tabooing unconditional but enabled by *stipulated positioning* as in soft laws (palliative care guidelines) and hard laws (euthanasia/PAS legislation). From a global perspective three shapes of dying control emerge. First, suboptimal palliative care in closed awareness contexts seen in Asian, Islamic and Latin cultures, called *closed dying*. Second, palliative care and sedation therapy, but not euthanasia or PAS, is seen in Europe and North America, called *open dying with reversible medical control*. Third, palliative care, sedation therapy, and PAS or euthanasia occurs together in the Benelux countries, Oregon, Washington and Montana, called *open dying with irreversible medical control*.

**Conclusions:**

De-tabooing dying control is an assumed secular process starting with open awareness contexts of dying half a century ago, and continuing with the growth of the palliative care movement and later euthanasia and PAS legislation.

## Background

For centuries the subject of dying has been a taboo in many cultures [1], where a taboo is an inhibition or ban on saying or doing something that results from social custom or emotional aversion according to dictionary definitions. Therefore it has been difficult to openly discuss issues about death and dying when it comes to how we want to die. The systematic killing of patients referred to as ‘useless eaters’ in Germany during the Nazi era has, since the Nuremberg Trials, associated euthanasia with killing patients for financial or ideological reasons. This has contributed to a strong taboo against euthanasia in many countries, particularly in Germany [2], which applies strict end-of-life legal policies [3].

In the 1960s Glaser and Strauss studied the American “taboo of death” in the transformed social-psychological landscape of the twentieth century [4]. They researched what was going on regarding death and dying in American hospitals [5]. Different types of awareness contexts explained the attitudes and actions among terminally ill patients, relatives and health care professionals; different people exhibited different behaviours depending on who knew what about the nature of the patient’s dying and the likely timing of the death. In a closed awareness context, persons are unaware of their imminent death and hospital staff strives to hide this knowledge. In an open awareness context persons are aware of dying bringing a new set of needs to the dying situation requiring different responses by staff. In those dying situations where both patient and staff are aware but pretend not to know, both parties engage in what is termed a ‘ritual drama of mutual pretense’, suggesting a role play in the dying situation [6]. One argument for closed awareness at that time was that a terminally ill patient, if made aware of the prognosis, might commit suicide. Indeed, the relative suicide rate is higher among cancer patients [7,8] and patients with incurable neurodegenerative diseases such as ALS and Huntington’s disease [9,10] as compared to the general population, with the highest suicide rates early in the disease trajectory [8,11,12]. Evidently, open awareness contexts also raise the patient’s awareness of the option of ending one’s life in a controlled manner.

The purpose of the present study was to analyze what is going on in the field of dying control, and eventually to present a grounded theory of control of dying focusing specifically on how people react in relation to issues about euthanasia and PAS.

## Methods

### Data collection

The first author (HT) collected data from interviews with 55 laypersons, nurses, and physicians from Belgium, Canada, Denmark, Ireland, Netherlands, Norway, Sweden, UK, and the USA from 2007 to 2010. The interviews were colloquial conversations carefully recorded in field notes in line with the classic grounded theory dictum “all is data”, see below. The interviews mostly took place in public places, conferences, professional workshops, but also in private settings. Four Internet forums discussing euthanasia and PAS from 2002–2007 were analyzed using the same classic grounded theory method as described below, with a total of 40 participants from the USA and Canada making 160 unique postings. Scientific and popular literature on PAS and euthanasia was also studied in the theory generation process including articles and comments from the press, radio and television. Most of the scientific literature compared is found in the reference list of this paper.

We also collected data from two similar postal surveys with the same 15 multiple-choice items regarding end of life issues in 2007. 1200 physicians were randomly selected for the first postal survey, while the second survey was distributed to 1200 randomly selected individuals living in the County of Stockholm, Sweden. The purpose was to investigate attitudes towards PAS. The response rate was 58% in the population and 74% for physicians. Almost 30% of the public and physician respondents gave open-ended written comments amounting to a total of 6500 words. Respondents older than 50 years provided more comments or free text statements and were more in favor of PAS as compared to younger participants. The commentary survey data in this study has been presented elsewhere using qualitative content analysis [13].

### Data analysis

Data was analyzed using the classic grounded theory method according to Glaser and Strauss and Glaser [14-21] applying the same procedures as in recent studies [22,23]. Classic grounded theory uses an inductive approach to generate hypotheses which explain how participants in a studied substantive area resolve their main concern. Classic grounded theory aims at generating conceptual theories abstract of time, place and people. Classic grounded theory studies differ from studies using qualitative data that claim to be grounded theory by presenting explanatory concepts rather than descriptions. All of the data mentioned above was compared in the analysis according to the classic grounded theory” all is data” dictum [15-17]. The literature was also analyzed based on classic grounded theory principles. The literature review was thus not done until the core concepts of the theory had emerged through a cyclic process of collecting, coding, and comparing incidents in the data. Theoretical memos, in the shapes of text, diagrams, and figures, were written, typed, or drawn and several hundreds of pages of typed and handwritten memos sit in the memo bank from which this paper was sorted and written up. ”Memos are the theorizing write-up of ideas about substantive codes and their theoretically coded relationships as they emerge during coding, collecting and analyzing data” [17]. A classic grounded theory research conceptualizes “what is going on” in the field of study by constantly comparing data during an iterative research process which involves open coding, memoing, theoretical sampling (=data collection based on emerging hypotheses from the ongoing analysis), selective coding (=coding and recoding particular data based on central concepts from the ongoing analysis), sorting (=sorting memos according to relationships between concepts in the theory) and re-sorting and then writing up the sorted memos into a paper or book. Authors HT and HS have read and reread the method literature and participated in and led numerous classic grounded theory trouble shooting seminars. The authors have also worked to discipline their subjectivity in order to stay open to what emerged in the data. Once the core category explaining what was going on in the data (=de-tabooing) was generated, the analysis was delimited to de-tabooing and related categories and selective coding was done. Memoing with de-tabooing guiding the analytic work then pursued. Eventually, facilitated by theoretical sorting and writing up, an integrated set of hypotheses emerged with the aim of explaining the main concern of participants of the studied area. As for the cycling of the data analysis the first data from this study came from informal talks with physicians and researchers in 2001. Then followed the survey data analysis and thereafter more informal interviews with lay people and health care professionals. Eventually the internet forums were analyzed and finally the literature. In the Appendix is presented one early and one late memo from the first author.

Many clinical research methods consider persons or patients as units of analysis, whereas in classic grounded theory the unit of analysis is the incident not the person(s) involved (where an incident is a distinct piece of action or an episode). The number of incidents being coded and compared typically amounts to at least several hundred in a classic grounded theory study since every participant often reports many incidents. The data in this study amounts to many more than one thousand incidents since the surveys had almost 1500 respondents and almost 500 survey respondents provided qualitative comments. A classic grounded theory study does not produce data based on a pre-existing theory, but generates hypotheses for a new theory based on a thorough and systematic analysis of a large amount of data, both empirical and interpreted, quantitative as well as qualitative. Classic grounded theories are thus not reports of facts but integrated conceptual hypotheses based on empirical data. The quality of a classic grounded theory may be tried against the principles of fit, work, relevance and modifiability set forth by Glaser and Strauss [14] and Glaser [15,17]. Fit has to do with how closely concepts fit the incidents they are representing, and achieving ‘fit’ requires rigorous adherence to the constant comparison process, where incidents are compared to each other and to emerging concepts. A relevant study deals with the real concern of participants and captures attention. The theory works when it explains how the problem, or main concern of participants, is being solved and when it accounts for most of the variation in participants’ behaviors in the substantive area. A modifiable theory is one that is never ‘finished’ and can always be developed as and when new relevant data is compared to existing data. A classic grounded theory is never right or wrong, it just has more or less fit, relevance, workability and modifiability, and readers of this paper are asked to assess its quality according to these principles.

Neither the mail survey nor the interview data in this study required formal research ethics approval according to Swedish law, but the Regional Research Ethics Committee in Stockholm gave a positive advisory statement regarding the survey directed to the public (Diary number 2007/310-31). Conceptual [13] and descriptive data [24] from the survey part of this study has been reported previously.

## Results

### De-tabooing dying

In this study, based on conceptualized data using classic grounded theory, it is proposed that a de-tabooing process can explain much of what is going on regarding issues of medically controlled dying in the western world of today. De-tabooing contains a struggle between the taboo of dying control and an emergent taboo against questioning autonomy and self-determination of healthy people and patients that is called de-paternalizing.

De-tabooing dying control started with open awareness of dying. In the 1960s, when Glaser and Strauss discovered different awareness contexts of the dying situation in six American hospitals, medical paternalism was routine in the patient-physician relationship [5]. Over the last forty years, however, the death and dying discourse has changed. Paternalism is increasingly difficult to defend when treating competent patients and has eventually become a dysphemism, indicating an emergent taboo of questioning autonomy. The open awareness context of the dying situation, today a norm in many Western societies, is the starting point for a control of dying that can be met by palliative care, euthanasia or PAS [25-27]. Open awareness contexts are associated with a higher demand for hospice care, and also with a wish for euthanasia, another indicator of the desire to control the manner and timing of death [27].

A taboo positioning reaction was observed in our data both at the individual and the macro level. This illustrates the tension between the dying control taboo and the emergent taboo against the questioning of patient autonomy and self-determination.

### Taboo positioning

A positioning resulting from the interaction between the weakening taboo connected with euthanasia and PAS and an emergent taboo against the questioning of patient autonomy and self-determination was identified. Between these two tendencies there are power and values at stake dealing with the question “who should ultimately control dying?” Should it be the health care staff including physicians or should it be the patients themselves?

When people engage with the issue of who should control dying, there is often an immediate *emotional positioning* for or against euthanasia and PAS. Emotional positioning involves conceptual confusion since many respondents did not make a distinction between euthanasia, PAS, sedation therapy, and withdrawing or withholding life-sustaining treatment. Then follows *reflected positioning*, where the emotional reaction is reflected upon and sometimes modified. Participants might also engage in a *labeling wrestle* using euphemisms and dysphemisms. If one side in the interaction struggle gains dominance, there will be *stipulated positioning*, including ethical reasoning and soft laws (e.g. palliative care guidelines). This is sometimes formalized in hard laws (e.g. euthanasia and PAS legislation) and followed by new behaviors of *defending the new norm* when it is almost an anathema to question patient autonomy.

To expound: *emotional positioning* is an emotionally triggered non-cognitive reaction resulting in a positive or a negative attitude to the issue of the control of dying. A first reaction to the taboo is thus an emotional “gut-feeling” response and emotional positioning therefore takes place before the taboo issue is fully defined. This pattern of immediate emotional positioning, seemingly without previous cognitive reflection, was seen over and over again both in the survey data and in the interviews. Emotional positioning could also include a reaction against the tabooing of questioning patient autonomy.

*Conceptual confusion* is rife; euthanasia, PAS, and withdrawing life sustaining treatment are not seen as separate entities, and people confuse the concepts they are positioning themselves toward. Although we did not ask about withdrawal of life sustaining treatment, many survey and interview respondents answered as if this was synonymous to euthanasia and PAS.

“Conceptual confusion is always present when it comes to death and dying. There is never clarity when issues in these areas are discussed.”

(Swedish physician interviewee)

### Reflected positioning

Emotional positioning is followed by a reflecting phase involving reasoning and examination of pros and cons by exemplifying and comparing. Reflected positioning thus involves a cognitive juggling of the control of dying taboo by reasoning for and against it. A similar wrestling with the taboo towards questioning patient autonomy takes place. In this way the position taken is defended and explained.

“You take on an attitude based on emotions and after that you construct a theory that defends that position.”

(Swedish nurse interviewee)

However, some persons may actually change their attitudes in the reflective process or go from a definite position to a more hesitant one.

“When I come to think of it (euthanasia), it is probably not good to legalize it even if I would like it for myself.”

(Swedish public survey respondent)

Survey response examples of arguments in favor of PAS were that it is inhumane to leave some patients in unbearable suffering and that offering PAS would be a way to respect patient autonomy. Counter arguments to PAS concerned risks of abuse, the slippery slope, procedural failures, and professional role erosion. It was also argued that euthanasia and PAS are not needed as means to pain relief if proper palliative care and sedation therapy is offered [28].

### Labeling wrestle

Taboos are often labeled by dysphemisms, and that calls for euphemisms to replace them in the de-tabooing process. If “murder” is a dysphemistic labeling of euthanasia, then “dignified death” is a euphemistic label of the same incident. Participants thus argue with euphemisms or dysphemisms that label PAS as good or bad during taboo positioning. When different types of controlled dying are labeled in dysphemistic terms this often results in a euphemistic countering and vice versa. For example, the Swedish word for suicide is, literally translated, “self-murder” and might thus be understood as a dysphemism, and several survey respondents requested a more neutral expression.

“(It is) wrong to use the negatively loaded words murder and suicide.”

(Swedish physician survey respondent)

“I would prefer a nicer word than suicide.”

(Swedish public survey respondent)

The labeling wrestle may also be part of defending new norms as discussed below.

Stipulated positioning occurs when an individual transcends the taboo but the transcendence is conditionalized. There are patterns to the arguments of stipulated positioning, for example it is commonly stipulated that PAS is acceptable when proper protection is in place to protect individuals against the abuse of laws permitting medically controlled dying; that adequate safety and legal criteria are met which protect both the patient and those assisting dying. It is often argued that autonomy and self-ruling is essential and medically controlled dying must be something the patient wants for him or herself; and the decision should not be influenced by others.

“It (PAS) is OK if the law requires that it is the definite wish of the patient.”

(Swedish physician survey respondent)

It was often argued by participants that the patient must suffer from an incurable deadly disease and have a short life expectancy. Yet, mentally capable and paralyzed individuals are challenging society and current legal structures for the right to a medically controlled dying without repercussions for those assisting. Ultimately for social acceptance, the control of dying has to be approved by legal authorities and monitored by skilled physicians. Thus legal stipulating by changing the law is a powerful indicator of transcending the taboo of medically controlled dying in any society.

“This is what happens when we transcend a taboo. Then we need to regulate it with great precaution”

(Dutch Law professor explaining why the Dutch euthanasia law is so detailed)

Thus, stipulated positioning eventually signals societal acceptance of changes to the law governing medically controlled dying. Euthanasia became common in the Netherlands in the 1970s after it was de-penalized [29-31]. In 2001 euthanasia was de-criminalized in Dutch law under certain conditions and similar laws followed in the neighboring countries of Belgium (2002) and Luxembourg (2008). PAS was made legal in Oregon, USA, in 1997 and in the neighboring states of Washington and Montana in 2008. The WHO definition of palliative care and different national and international guidelines for palliative care and sedation therapy seem to function as ‘soft laws’. In this way palliative care is a part of de-tabooing medical control of dying.

### Defending the new norm

When the taboo of euthanasia and PAS has been legally transcended with liberal laws, as in the Benelux, Montana, Oregon and Washington, many people are defending the new norm simply because to question it has become a new taboo. A Dutch physician observed that “People now see it as a human right” and to question human rights is taboo in a democratic society. Criticizing euthanasia and PAS might thus be understood as a re-tabooing attempt while defending the new norm is a de-tabooing activity.

### Closed or open dying

Three different positions in the control of dying discourse were identified. Those dying situations where there is a low level of awareness of the imminent death and a low degree of medical control of dying is a pattern labeled ‘closed dying’. Two further types of dying situations exhibiting open awareness of an imminent death exist; one with a reversible control of dying, called “open dying with reversible medical control” and another with irreversible control of dying: “open dying with irreversible medical control”.

#### Closed dying

In situations with a low level of awareness of dying and little medical control of dying there may still be palliative care, but it is generally agreed that optimal palliative care services require an open awareness context [32]. In a closed awareness context, it is not possible to discuss either reversible or irreversible control of dying, and patient autonomy regarding end-of-life decisions is consequently low. This pattern is mostly observed in Asian, Islamic and Latin cultures [33-36].

#### Open dying with reversible medical control

When physicians discuss death and dying more openly, they tend to associate the discussion with palliative care including, as a last resort, sedation therapy. Although critics have referred to sedation therapy as slow euthanasia, it has been formally accepted in a medical context under certain conditions [37-39]. Palliative care is increasingly important in health care but still has low priority at medical schools and in nursing education. This may be seen as a consequence of the remaining control of dying taboo. Although most people see palliative care as entirely different from euthanasia and assisted suicide, some do not separate palliative care from euthanasia and PAS [40].

#### Open dying with irreversible medical control

In the general population an open discussion of the medical control of dying also encompasses discussion of the methods used in the irreversible control of dying. These methods include withholding life support treatment or ending such treatment, which is legal in most western countries. Yet, more controversial is irreversibal controlled dying by euthanasia and PAS, which is illegal in most countries. In the media the issue has repeatedly been discussed when patients suffering from terminal diseases travel to Switzerland, where assisted suicide has been legal since 1918 [41].

Public and private debate of both reversible and irreversible medical control of dying is part of the de-tabooing dying trend. Reversible control of dying includes openness and awareness of dying to provide adequate symptom relief, but the traditional palliative care position does not embrace sedation therapy at the patient’s request. The reversible medical control of dying attitude of traditional palliative care is therefore more inclined to support paternalism than to defend the taboo of questioning autonomy [42].

## Discussion

De-tabooing dying control is the suggested label for an ongoing secular pattern in the Western world enabling and resulting in structural changes in end-of life care. De-tabooing dying control started in the open awareness contexts of hospitalized dying which emerged half a century ago and explains the growth of the palliative care movement and later the development of euthanasia and PAS which thus are seen as results of a de-tabooing process. De-tabooing dying control involves a tabooing of the questioning of patients’ self-determination and autonomy, a nested de-tabooing sub process called de-paternalizing. De-tabooing involves verbal strategies such as using euphemisms and dysphemisms to differentiate stances taken in a taboo positioning process that usually begins with an emotional reaction including cognitive confusion followed by cognitive reasoning. Finally, a tendency of stipulating both soft and hard laws and defending the new norm appears.

Three de-tabooing sub-positions emerged – closed dying, open dying with reversible medical control and open dying with irreversible medical control. Euthanasia and PAS are still taboo within the open reversible dying position, which instead emphasizes palliative care and eventually sedation therapy.

The classic grounded theory analytic approach according to Glaser [14-21] was used to develop the de-tabooing process model. The procedure was similar to that used in previous studies in other substantive areas and is presented in detail elsewhere [22,23]. Classic grounded theory is the most cited single method for analyzing qualitative data according to Google Scholar where the first grounded theory methodology “Discovery of Grounded Theory” [14] had 47605 citations in December 2012. Yet classic grounded theory studies are rare. They represented <10% of 200 consecutive studies referring to the grounded theory method in a PubMed search in 2005–2006 [23]. Most of these studies were descriptive and lacked a core variable theory, which is required in classic grounded theory.

Kübler-Ross in 1969 presented her five stage process of dying in the popular book “On Death and Dying” [43]. This process can be seen as another de-tabooing path after the Awareness of dying theory by Glaser & Strauss [5]. Kübler-Ross dealt with a taboo topic by generating a logical theory that gave a sense of controlling the taboo [44]. De-tabooing dying converges with the grounded theory of balancing end-of-life care [45,46]. Balancing and de-tabooing both deal with end-of-life and how to approach it. Balancing explains problem-solving strategies of health care staff and physicians and offers a comprehensive perspective on end-of-life care and how dying is controlled between cure and comfort care. The balancing outcome is characterized by compromising, at best an optimized situation, at worst a deceit. De-tabooing control of dying explains attitude and legal changes regarding end-of-life and involves different types of positioning between the taboos of control of dying and paternalism. Compromising is a property of positioning, which in turn is an important part of balancing between the many different care options at the end-of-life.

A suicide researcher suggested a de-tabooing of the suicide discourse after the start of a zero-vision program re suicide with increased resources for education and research [47,48].

De-tabooing goes on in other substantive areas as well. A literature search shows many sociological researches on de-tabooing sexuality in film, literature and advertisement with movie directors Pedro Almodovar and Bigas Luna representing deta-tabooers [49]. A more restrictive immigration policy in Western Europe made it less taboo to criticize immigrants and thus vote for a right wing party which was seen as a de-tabooing process partially explaining the growth of right wing voting in Europe these last decades [50].

### Limitations

A main limitation of this study is that we only used population survey data from Sweden. Yet, the constant comparison of the grounded theory method compensates for particularistic bias since relevant grounded theory concepts are abstract of time, place and people. The different categories that emerged from attitude patterns in the Swedish survey data were constantly compared and carefully fitted with international interview data, American discussion forum data, literature [51], and news data. This leads us to conclude that the Swedish survey data was rich enough to allow conceptualizations that are relevant to other cultural settings as well.

The attitudes of the public in the survey data from this study came from a sample of people living in Sweden’s largest urban area. While it is known that euthanasia is twice as common in Dutch urban parts as in rural areas [52], had our sample also involved rural areas, the views might have been less positive to PAS. But, also this bias is of small importance in a thorough grounded theory.

Most of the physician data in this study came from Sweden. It therefore must be said that Swedish physicians had much stronger life preserving attitudes than their colleagues in Belgium, Denmark, Netherlands, Switzerland and Australia in a large comparative study on physicians’ end-of-life views [53]. The fact that the first and last authors (HT and NL) of this study both are Swedish physicians may therefore explain why the taboo concept emerged as central in this study.

One may argue either that dying has never been de-tabooed or that control of dying has never been a real taboo topic. Yet the reactions of emotional positioning and the cognitive reflecting, labeling and stipulating, were seen in large numbers in our interviews, survey, Internet forum, and news data. The immediate emotional reaction to a taboo seems to be part of a normal psychological reaction where attitude processes start with an emotion. This observation is well supported by neuroscience findings and theories emphasizing the importance of emotions for decision-making [54-56], including moral judgment [57]. Finally, there is literature support for the de-tabooing control of dying proposition that open awareness of dying has paved the road for a control of dying that can be met by either euthanasia and assisted suicide or palliative care [25-27].

## Conclusions

De-tabooing dying control emerged as a pattern of behavior in the substantive area of dying in the Western world starting with open awareness contexts of dying half a century ago, and continuing with the growth of the palliative care movement and later euthanasia and PAS legalization in a growing number of jurisdictions. De-tabooing dying control can be observed both on the level of how individuals momentarily react to dying control taboos and how groups of people respond over longer periods of time.

## Appendix

Memo example 1. Early de-tabooing memo relating to the survey responses

“Paternalistic perspectives versus autonomic perspectives. In a paternalistic perspective we are responsible for others and take decisions in their place. This is something the population knows and therefore they like being taken care of by the paternalist – the God like physician. They trust the physicians’ competence and do not have to take responsibility for something as distasteful as to end life. Physicians are meant to take care of life and death and difficult decisions involving this. There was a pattern among physicians of certain specialties – also differences between women and men and younger and older physicians. So, PAS combines paternalism with autonomy? Or rather the paternalism works in favor of what the patient wants? Autonomy is when the patient gets what he/she wants and medical paternalism is when the patient gets what the physicians wants her to have. 2007 April”

Memo example 2. Later de-tabooing memo relating to the literature

“De-tabooing – re-tabooing. Re-tabooing is an attempt, sometimes semantically by using dysphemisms, to defend the taboo. To be against euthanasia is a useful taboo according to a German dr. Germany is a culture with a strong anti-euthanasia discourse. Probably caused by the severely negative load of the euthanasia word inherited from the Nazi era. Ie that word is such a powerful dysphemism that it strongly controls the way people react to the taboo. Maybe we here in Sweden also have a strong dysphemistic load against the concept of euthanasia. And this may come from our liaison with the Nazi discourse that went on in the 1950s with eugenics performed to not only mentally disabled, but also promiscuous women were sterilized against their will. Medical paternalism ruled, and this is nothing we want back. 2010 July”

## Competing interests

The authors declare that they have no competing interests.

## Authors’ contributions

HT, GH and NL participated in research design. HT conducted interviews, did the basic grounded theory analysis and drafted the manuscript. All authors, HT, HS, GH and NL participated in analysis and writing and revised the manuscript for major intellectual content. All authors read and approved the final manuscript.

## Pre-publication history

The pre-publication history for this paper can be accessed here:

http://www.biomedcentral.com/1472-684X/12/13/prepub
